# An Analysis of Recent Tetanus Statistics

**Published:** 1918-01

**Authors:** 


***An Analysis of Recent Tetanus Statistics.*** By F. Golla, Capt. R. A. M. C. (T. F.). Abs. from the *Lancet*, Dec. 29, 1917.

G. summarizes all the results obtained in the British army for the years 1916 and 1917, so far as these are available. In all 640 cases have been reported. The total mortality was 50.8 o/o. G. thinks this low mortality due mainly to the protective injection of serum, and thinks the percentage encouraging when compared with percentages available in the military statistics for pre-serum days. Most of the groups reported for earlier wars, notably that of the Secession, the Franco-Prussian war, and the Crimean war, have a recorded mortality of about 90 o/o.

As to curative treatment by serum, G. finds no certain progress indicated by the statistics so far available. He notes particularly that in man, unlike animals observed in experiments, there is no correspondence whatever between the length of time elapsed before the administration of the serum and the results obtained by treatment. Similarly there is little evidence that the amount of serum administered makes any appreciable difference in the results obtained.

In reviewing the work so far done experimentally on animals, G. notes that clinical evidence does not support the encouraging results obtained by laboratory experiment. It is at any rate known that if a sufficient amount of toxin has already entered the central nervous system of the animal, there is little possibility of neutralizing it by an injection of serum. The failure of serum in man, therefore, is probably due to the fact that when the serum is administered, curatively, there is already enough toxin in the nerves to cause death in any case. Likewise, in the mild cases that recover, there is probably not enough toxin developed at any time to cause death. Another reason for the failure of serum in man, the author thinks, may be the inflamed condition of the tissues about the wound, which prevents easy access of the antitoxin to the toxin. In the animals, on the other hand, the toxin lies loose in the tissues. G., therefore, believes that animal experiments must be begun again, reproducing the septic wound condition as well as the infection with tetanus.

